# ZNF121 interacts with ZBRK1 and BRCA1 to regulate their target genes in mammary epithelial cells

**DOI:** 10.1002/2211-5463.12530

**Published:** 2018-11-08

**Authors:** Ang Luo, Kailun Zhang, Yanxia Zhao, Zhengmao Zhu, Liya Fu, Jin‐Tang Dong

**Affiliations:** ^1^ Department of Genetics and Cell Biology Nankai University College of Life Sciences Tianjin China; ^2^ Department of Hematology and Medical Oncology Winship Cancer Institute Emory University School of Medicine Atlanta GA USA; ^3^ Present address: Department of Biochemistry, Molecular Biology and Biophysics University of Minnesota‐Twin Cities 420 Washington Avenue SE Minneapolis MN 55455 USA

**Keywords:** BRCA1, ZBRK1, ZNF121

## Abstract

The novel zinc finger protein 121 (ZNF121) has been demonstrated to physically and functionally associate with the MYC oncoprotein to regulate cell proliferation and likely breast cancer development. To further understand how ZNF121 functions in cell proliferation and carcinogenesis, we identified and characterized the interaction of ZNF121 with zinc finger and BRCA1‐interacting protein with a KRAB domain 1 (ZBRK1), a breast and ovarian cancer susceptibility protein 1 (BRCA1)‐interacting protein, using the yeast two‐hybrid assay and other approaches. We also found that ZNF121 bound to BRCA1. Functionally, ZFN121 suppressed the expression of ANG1 and HMGA2, two common downstream targets of ZBRK1 and BRCA1. Interestingly, ZNF121 also regulated the expression of BRCA1 and ZBRK1. These findings suggest that ZNF121 is likely a member of the BRCA1/CtIP/ZBRK1 repressor complex that plays a role in breast cancer.

**Abbreviations**
ANG1: angiopoietin‐1
BRCA1: breast and ovarian cancer susceptibility protein 1
Dox: doxycycline
GADD45α: growth arrest and DNA‐damage‐inducible 45 alpha
HMGA2: high‐mobility group AT‐hook 2
MMP9: matrix metallopeptidase 9
ZBRK1: zinc finger and BRCA1‐interacting protein with a KRAB domain 1
ZNF121: zinc finger protein 121

As one of the largest families of regulatory proteins in human cells, the Cys_2_‐His_2_ (C2H2) zinc finger proteins have been reported to play important roles in development, differentiation, and human diseases 1. However, due to their large numbers in the human genome, most of the zinc finger proteins are not well studied. The ZNF121 zinc finger protein is one such factor. A large‐scale combination of tandem affinity purification with the mass spectral multidimensional protein identification technology (MudPIT) suggests that Zinc finger protein 121 (ZNF121) interacts with the MYC oncoprotein 2. MYC has been demonstrated to be one of the most potent oncoproteins that participates in a broad range of cellular processes, such as cell proliferation, differentiation, stemness, apoptosis, cell migration, and metabolism 3, 4 to regulate multiple aspects of tumorigenesis 5.

In our recent study, we used the yeast two‐hybrid assay and other approaches to confirm that ZNF121 indeed interacts with MYC 6. In addition, ZNF121 and MYC regulate each other's protein expression or stability, and ZNF121 indeed regulates cell proliferation, apoptosis, and likely breast cancer development 6. The *ZNF121* mRNA level is also higher in breast cancer samples compared to normal tissues. These findings suggest that ZNF121 likely plays an oncogenic role in breast cancer, yet very little has been reported about any aspects of ZNF121 in the literature.

In this study, we continued to characterize ZNF121‐interacting proteins in the context of breast cancer development. A yeast two‐hybrid assay was performed to identify the zinc finger and BRCA1‐interacting protein with a KRAB domain 1 (ZBRK1) as a new interacting partner of ZNF121. Previous studies have demonstrated the interaction between ZBRK1 and the breast and ovarian cancer susceptibility protein 1 (BRCA1) breast cancer molecule in the transcriptional regulation of many BRCA1 target genes, including growth arrest and DNA‐damage‐inducible 45 alpha (GADD45α), a protein involved in cell cycle control and DNA damage response 7, 8, angiopoietin‐1 (*ANG1*) 9, 10, and high‐mobility group AT‐hook 2 (*HMGA2*) 11, 12, 13. The regulation of these genes depends on the binding of ZBRK1 to the canonical GGGxxxCAGxxxTTT motif on their promoters. We therefore further tested and verified the interaction between ZNF121 and ZBRK1 in mammalian cells. Interestingly, BRCA1 was also shown to bind with ZNF121. Similar to ZBRK1 and BRCA1, ZNF121 also modulated the expression of *ANG1* and *HMGA2*. ZNF121 also affected the expression of ZBRK1 and BRCA1.

## Materials and methods

### Yeast two‐hybrid assay

Yeast two‐hybrid assay was conducted with the Matchmaker Gold Yeast Two‐Hybrid System (Clontech, Mountain View, CA, USA). The full‐length CDS sequence of ZNF121 was cloned into pGBKT7 vector and transformed into the Y2HGold yeast strain. Mating was done with a universal human cDNA library in Y187 yeast strain. After mating, the yeast cells were seeded on SD/–Leu/–Trp/X‐a‐Gal/AbA (DDO/X/A) plates, and then, the positive colonies were transferred to SD/–Ade/–His/–Leu/–Trp/X‐a‐Gal/AbA (QDO/X/A) plates. Positive colonies growing on QDO/X/A plates were expanded for plasmid extraction, transformed into DH5α‐competent *Escherichia coli* cells, and then sequenced.

### Cell lines and drugs

Cell lines used in this study were all from the American Type Culture Collection (ATCC, Manassas, VA, USA). HEK293T cells were maintained in Dulbecco's Modified Eagle's medium (Gibco, Shanghai, China), supplemented with 10% FBS. T‐47D cells were cultured in RPMI1640 medium (Gibco) containing 10% FBS. MCF10A cells were cultured in F12/DMEM (Gibco) supplemented with 5% horse serum, 20 ng·mL^−1^ EGF, 0.5 mg·mL^−1^ hydrocortisone, 100 ng·mL^−1^ cholera toxin, and 10 μg·mL^−1^ insulin. Drugs used in this project included puromycin (P8833, Sigma, Shanghai, China), G418 (Genview, A‐138‐G), polybrene (H9268, Sigma), and doxycycline (D9891, Sigma).

### ELISA

ELISA was performed using the Human Angiopoietin 1 (ANGPT1) ELISA kit (DLDEVELOP, Wuxi, China) according to the manufacturer's instructions. Briefly, cells were seeded and transfected with siRNAs in 48‐well plates. Twenty‐four hours after transfection, the cells were supplied with fresh medium and grown for another 24 h. Then, the cell medium was used for determination of the amount of ANG1. The concentration of ANG1 in each group was normalized to the cell number, which was determined using the Cell Counting Kit‐8 (CCK‐8) (Dojindo, Beijing, China) as previously reported 14.

### Construction of stable cell lines overexpressing ZNF121

The coding sequence of *ZNF121* was cloned into pLVX‐AcGFP1‐C1 vector (Clontech) to generate pLVX‐AcGFP1‐ZNF121 plasmid. Lentivirus was produced in HEK293T cells by transfecting the cells with pLVX‐AcGFP1‐ZNF121 and the packaging plasmids psPAX2 and pMGD2. Twenty‐four hours after transfection, the cells were replenished with fresh medium, and another 24 h later, the cell medium containing the virus was filtered and used to co‐infect MCF10A cells with lentivirus containing rtTA in the presence of 8 μg·mL^−1^ polybrene. Twenty‐four hours after infection, the cells were cultured in normal medium containing 1 μg·mL^−1^ puromycin and 200 μg·mL^−1^ G418. Pooled stable cells were designated as conditional MCF10A cells and used for further study. The expression of ZNF121 was induced with 1 μg·mL^−1^ doxycycline (Dox).

### Co‐immunoprecipitation (co‐IP) and western blotting (WB)

Cells were seeded and transfected with Entranster‐D reagent (Engreen Biosystems, Beijing, China) in 60‐mm plates. Twenty‐four hours after transfection, the cells were lysed in ice‐cold lysis buffer (150 mm NaCl, 50 mm Tris/HCl pH 7.4, 1% NP‐40, 10% glycerin, 1 mm EDTA, cocktail protease inhibitor (Roche, Basel, Switzerland) with PMSF added immediately before use). After centrifugation, 10% of supernatant was reserved as the input and the remainder was incubated overnight either with anti‐FLAG M2 affinity gel (Sigma), anti‐c‐Myc magnetic beads (Thermo Fisher, Rockford, lL, USA), or agarose‐conjugated anti‐GFP (D153‐8, MBL International, Woburn, MA, USA). Then, the gel or agarose was washed with chilled lysis buffer 5 times, resuspended in 60 μL 1 × SDS sample buffer and boiled for 5 min, and chilled on ice and centrifuged, and the supernatant was used for SDS/PAGE. For endogenous IP, 293T cells were harvested in lysis buffer and incubated overnight with 3 μg BRCA1 antibody (14823S, Cell Signaling Technology, Danvers, MA, USA) or normal rabbit IgG at 4 °C, followed by incubation with Protein A/G Plus‐Agarose (sc‐2003, Santa Cruz, Shanghai, China) for 2 h. The agarose was when washed and eluted as above. After electrophoresis, the proteins were transferred to poly(vinylidene difluoride) membrane and blocked with 5% skim milk in TBST (TBS+0.1% Tween‐20). After blocking, the membrane was incubated with primary antibodies overnight and HRP‐conjugated second antibody for 2–3 h, washed with TBST for 3 × 6 min, and developed with WesternBright ECL HRP substrate (K‐12045‐D50, Advansta, Menlo Park, CA, USA). Primary antibodies used include the following: ZNF121 (ab58156, Abcam, Cambridge, UK), Myc‐tag (c3956, Sigma), FLAG‐tag (F7425, Sigma), GFP (sc‐8334, Santa Cruz), and BRCA1 (sc‐6954, Santa Cruz).

### Immunofluorescence staining

293T cells were seeded on 12‐mm glass coverslips in a 24‐well plate at about 30% confluence. Twenty‐four hours later, cells were cotransfected with FLAG‐ZNF121 and Myc‐ZBRK1 plasmids, and the medium was replaced 12 h after transfection. Twenty‐four hours after transfection, cells were washed with PBS, fixed with 4% paraformaldehyde for 30 min at room temperature (RT), permeabilized with 0.5% Triton X‐100 for 15 min at RT, and blocked with 2% BSA for 30 min. Cells were then incubated simultaneously with FLAG‐tag antibody and Myc‐tag antibody at 4 °C overnight. After washing with PBS 3 times, cells were incubated with FITC‐labeled goat anti‐mouse IgG and TRITC‐labeled goat anti‐rabbit IgG at RT for 2 h. Nuclei were stained with DAPI, coverslips were mounted, and photographs were taken with a Zeiss LSM710 laser microscope.

### Plasmids and siRNA transfection, quantitative real‐time PCR (qRT‐PCR) and immunofluorescence

Details for these assays were the same as previously reported 6. The CDS sequence of ZBRK1 was obtained by PCR from the total cDNA of 293T cells and cloned into other vectors, including pGADT7, pCI‐Myc, pEGFP‐C1, and pCMV‐tag2b. pEGFP‐C1‐BRCA1 plasmid and the relevant truncation constructs were cloned from a pCMV‐tag2B‐BRCA1 plasmid that was used in a previous study 15. siRNA for ZBRK1 (siZBRK1, HSS184234) was purchased from Invitrogen (Carlsbad, CA, USA), and the sequence is as follows: 5′‐GAACUCUGUUGAGUUUACUGGAAAU‐3′. Sequences of primers for qRT‐PCR were as follows:*GAPDH*, 5′–GGTGGTCTCCTCTGACTTCAACA–3′ and 5′–GTTGCTGTAGCCAAATTCGTTGT‐3′; *ZNF121*, 5′–TTCGCCTTTATCGTGGTG–3′ and 5′–AATGTTGTTGAGGTGCTGAC–3′; *ZBRK1*, 5′–CCCAGTTGAATGCTGTCTTCC–3′ and 5′–CCACTCCTCCCAAGTGAAGTC–3′; *ANG1*, 5′–AGGTCACACTGGGACAGCAGGAA–3′ and 5′–CACAAGCATCAAACCACCATCCTCC–3′; *MMP9,* 5′–GCACTGCAGGATGTCATAGG–3′ and 5′– ACGACGTCTTCCAGTACCGA–3′; *HMGA2*, 5′–AAAGCAGCTCAAAAGAAAGCA–3′ and 5′–TGTTGTGGCCATTTCCTAGGT–3 ‘.

### ZNF121 shRNAs

The shRNA constructs for *ZNF121* were MISSION shRNA clones from Sigma. The sequences were as follows: shZNF121‐1 (TRCN0000117780), AACGTGGATAGGAGACAAA; and shZNF121‐2 (TRCN0000117779), CTTTGTTGATCAGTCACAT. Control shRNA plasmid encodes a scramble sequence that targets no genome sequence. Production of Lentivirus and selection of stable cell populations followed the same procedure as ZNF121‐overexpression in cells.

### Statistical analysis

Statistical analysis was conducted in Microsoft Excel. Student's *t*‐test was used to compare the differences between variables. *P* values < 0.05 were considered significant.

## Results

### Identification of ZBRK1 as an interacting protein of ZNF121

To explore how ZNF121 functions, we carried out a yeast two‐hybrid assay to screen for its potential interacting partners. Initial screening resulted in 39 positive colonies corresponding to 27 proteins, including ZBRK1, RNF2, ZNF17, ZNF200, ZNF198, and ZNF420. Besides ZBRK1, described below, RNF2 was confirmed to be a false‐positive colony (data not shown), and other proteins remain to be confirmed. The full list of potential ZNF121‐interacting proteins is shown in Table 1.

**Table 1:** List of potential ZNF121‐interacting proteins suggested by the yeast two‐hybrid screening

Name	Designations
ZNF23	Zinc finger protein 23 (KOX 16)
ZNF350	Zinc finger and BRCA1‐interacting protein with a KRAB domain 1
ZNF420	ATM and p53‐associated KZNF protein
RNF2	Ring finger protein 2
ZBTB38	Zinc finger and BTB domain containing 38
ZNF198	Homo sapiens Zinc finger, MYM‐type 2 (ZMYM2)
ZNF200	Zinc finger protein 200
ZNF438	Zinc finger protein 438
ARFGEF2	ADP‐ribosylation factor guanine nucleotide‐exchange factor 2
PAN2	Homo sapiens PAN2 poly(A) specific ribonuclease subunit homolog
VWF	von Willebrand factor
RFX5	Homo sapiens regulatory factor X,5
EAPP	Homo sapiens E2F‐associated phosphoprotein
SNX5	Homo sapiens sorting nexin 5
ZNF622	Zinc finger‐like protein
ZNF214	Zinc finger protein 214
ZNF17	Zinc finger protein 17
ZNF251	Zinc finger protein 251
ZNF302	Zinc finger protein 302
ZNF83	Zinc finger protein 83
ZNF561	Zinc finger protein 561
ZKSCAN5	ZKSCAN5 Zinc finger with KRAB and SCAN domains 5
PIFO	Primary cilia formation
SPATA22	Homo sapiens spermatogenesis associated 22
SPATA4	Homo sapiens spermatogenesis associated 4
GSG1	Homo sapiens germ cell associated 1
TSC21	Testis‐specific conserved protein of 21 kDa

**Figure 1:**
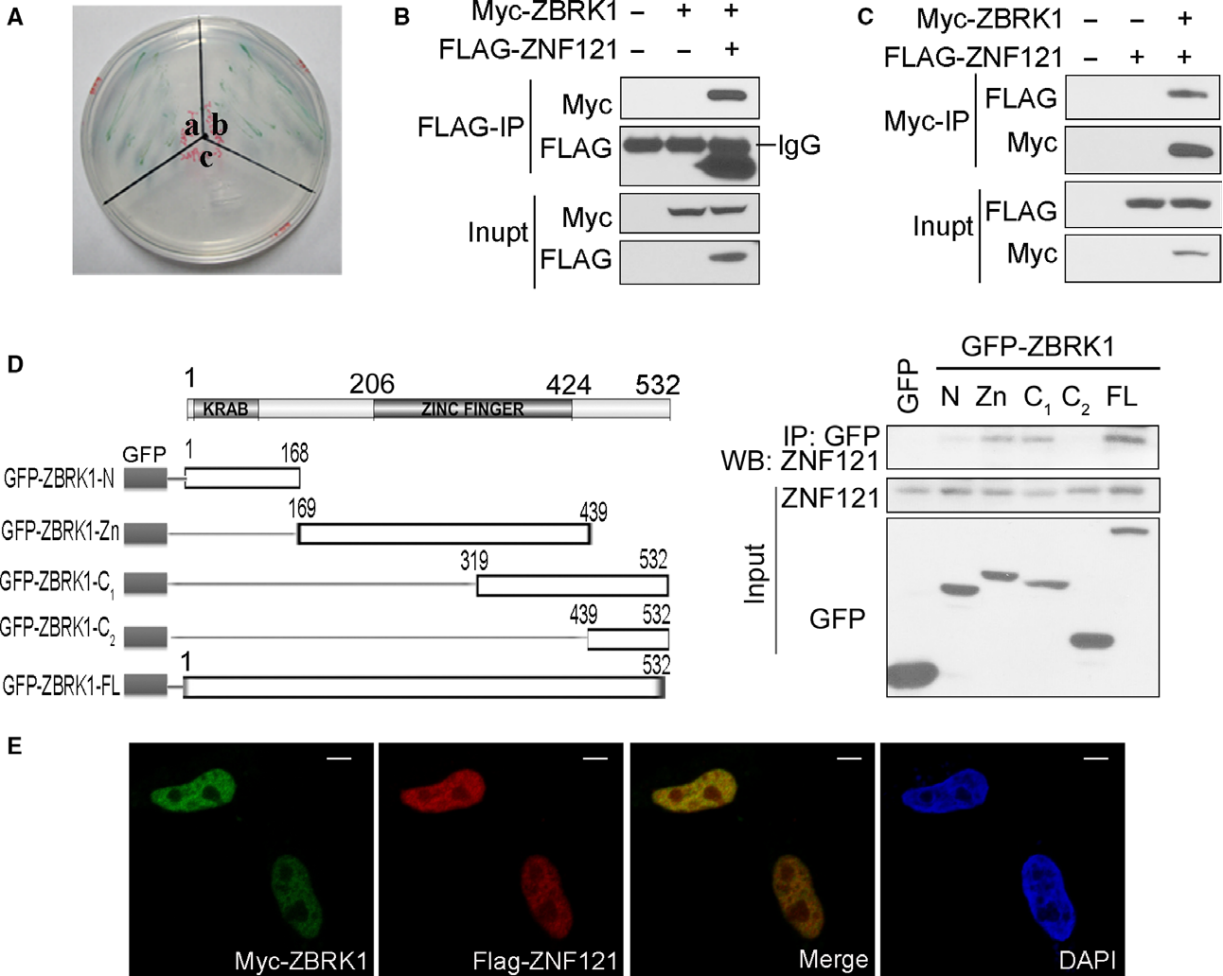
ZBRK1 interacts with ZNF121. (A) Demonstration of ZNF121′s interaction with ZBRK1 in yeast. Y2HGold yeast cells were respectively transformed with: a, pGBKT7‐p53+pGADT7‐T (positive control); b, pGBKT7‐ZNF121+pGADT7‐ZBRK1; and c, pGBKT7‐ZNF121+pGADT7 (negative control). Transformed yeasts were spread onto QDO/X/A plates and cultured at 30 °C for 4 days and then photographed. (B) FLAG‐ZNF121 interacts with Myc‐ZBRK1 in 293T cells. 293T cells were cotransfected with and pCI‐Myc‐ZBRK1 (Myc‐ZBRK1) and pCI‐FLAG‐ZNF121 (FLAG‐ZNF121). Twenty‐four hours later, the cell lysates were prepared for immunoprecipitation with FLAG antibody and WB was performed with Myc‐tag antibody. (C) 293T cells were transfected and analyzed as in B, but immunoprecipitation was done with Myc‐tag antibody. (D) Indicated ZBRK1 constructs (left) were respectively cotransfected into 293T cells together with Myc‐ZNF121 plasmid. Twenty‐four hours later, cell lysates were prepared for co‐IP with GFP antibody and WB with the indicated antibodies. (E) ZNF121 and ZBRK1 colocalize in 293T cells. Cells were transfected as in C, and then, immunofluorescence was performed and photographed with a Zeiss LSM710 laser microscope. Scale bars, 5 μm.

To confirm the positive interaction between ZNF121 and ZBRK1, a cotransformation assay was performed in Y2HGold yeast cells. Cells cotransformed with ZNF121 and ZBRK1 plasmids resulted in a signal comparable to the signal from the positive control cells. No signal was observed in the negative control cells, indicating an interaction between these two proteins in yeast cells (Fig. 1A). We then tested the interaction between ZNF121 and ZBRK1 in mammalian cells. FLAG‐ZNF121 plasmid was cotransfected into 293T cells with either Myc‐ZBRK1 plasmid or the control vector. Western blotting following co‐IP detected Myc‐ZBRK1 in the immunoprecipitates of FLAG‐ZNF121 (Fig. 1B). Consistently, FLAG‐ZNF121 was also detected in the immunoprecipitates of Myc‐ZBRK1 using the same approach, suggesting that FLAG‐ZNF121 and Myc‐ZBRK1 interact with each other in 293T cells (Fig. 1C). These data provide additional evidence supporting ZBRK1 as a new interacting protein of ZNF121.

To map the regions of ZBRK1 that mediate its interaction with ZNF121, we cloned different regions of ZBRK1 into the pEGFP‐C1 vector and directly tested the interaction of these truncated ZBRK1 proteins with the endogenous ZNF121 in 293T cells. Western blotting following co‐IP showed that GFP‐ZBRK1‐Zn, GFP‐ZBRK1‐C1, and full‐length ZBRK1 (GFP‐ZBRK1‐FL) were able to bind to ZNF121, suggesting that the zinc finger region of ZBRK1 is the key segment mediating its interaction with ZNF121 (Fig. 1D).

We also analyzed the cellular localization of these two proteins by immunofluorescence staining, and found that both Myc‐ZBRK1 and FLAG‐ZNF121 were localized in the nucleus, which suggests that their interaction occurs in the nucleus (Fig. 1E).

### ZNF121 interacts with BRCA1

**Figure 2:**
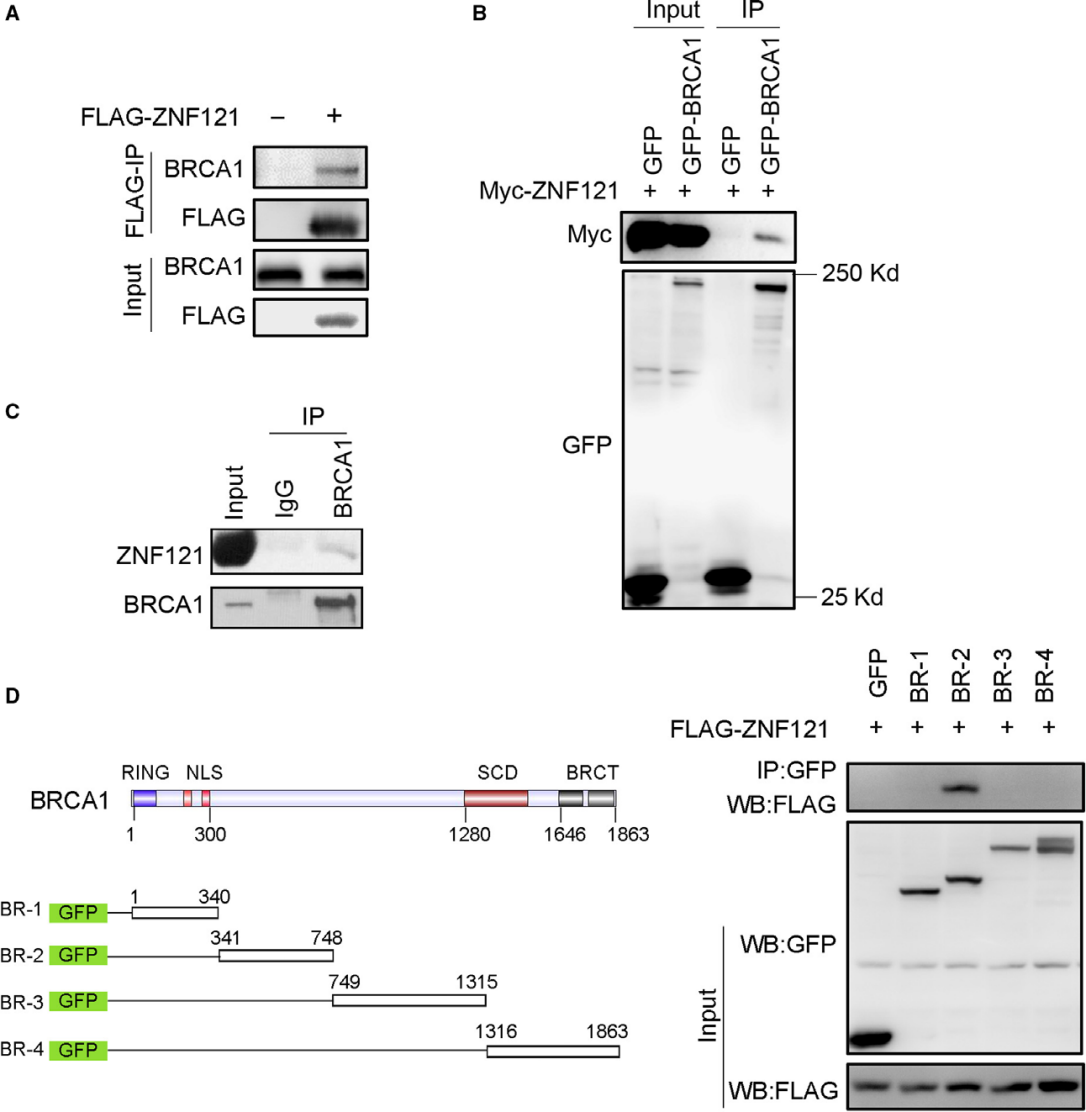
ZNF121 interacts with BRCA1. (A) Ectopically expressed ZNF121 interacts with endogenous BRCA1 protein in 293T cells. (B) Myc‐ZNF121 plasmid was transfected into 293T cells with either pEGFP‐C1 (GFP) or pEGFP‐C1‐BRCA1 (GFP‐BRCA1). Twenty‐four hours later, the cells were prepared for co‐IP with GFP antibody and then WB with the indicated antibodies. (C) Endogenous ZNF121 interacts with BRCA1 in 293T cells. (D) Mapping the regions of BRCA1 that bind ZNF121. Left: Schematic representation of different BRCA1 truncation constructs. Right: 293T cells were transfected with the indicated plasmids for 24 h, and then, co‐IP was performed with GFP antibody and WB with the indicated antibodies.

Considering that BRCA1 is one of the main known interacting proteins of ZBRK1 and that BRCA1 plays important roles in cellular processes and carcinogenesis, we tested the interaction between ZNF121 and BRCA1 in 293T cells. Western blotting clearly detected endogenous BRCA1 protein in immunoprecipitates pulled down by the FLAG antibody in cells transfected with FLAG‐ZNF121 but not in cells transfected with vector control (Fig. 2A). Similarly, Myc‐tagged ZNF121 was detected in the immunoprecipitates in cells transfected with GFP‐BRCA1 but not in cells transfected with GFP (Fig. 2B). In addition, endogenous ZNF121 was also detected in the immunoprecipitates of BRCA1 antibody (Fig. 2C). These observations suggest a physical interaction between ZNF121 and BRCA1 in 293T cells.

We mapped the regions of BRCA1 that mediate its interaction with ZNF121. Different regions of BRCA1 were cloned into the pEGFP‐C1 vector and co‐expressed with FLAG‐ZNF121 in 293T cells. Western blotting following co‐IP revealed that the BR‐2 region (amino acids 341–748), which mediates BRCA1′s interaction with ZBRK1 7, mediated the binding of BRCA1 to ZNF121 (Fig. 2D).

### ZNF121 regulates the expression of ANG1 in both MCF10A and T‐47D cells

**Figure 3:**
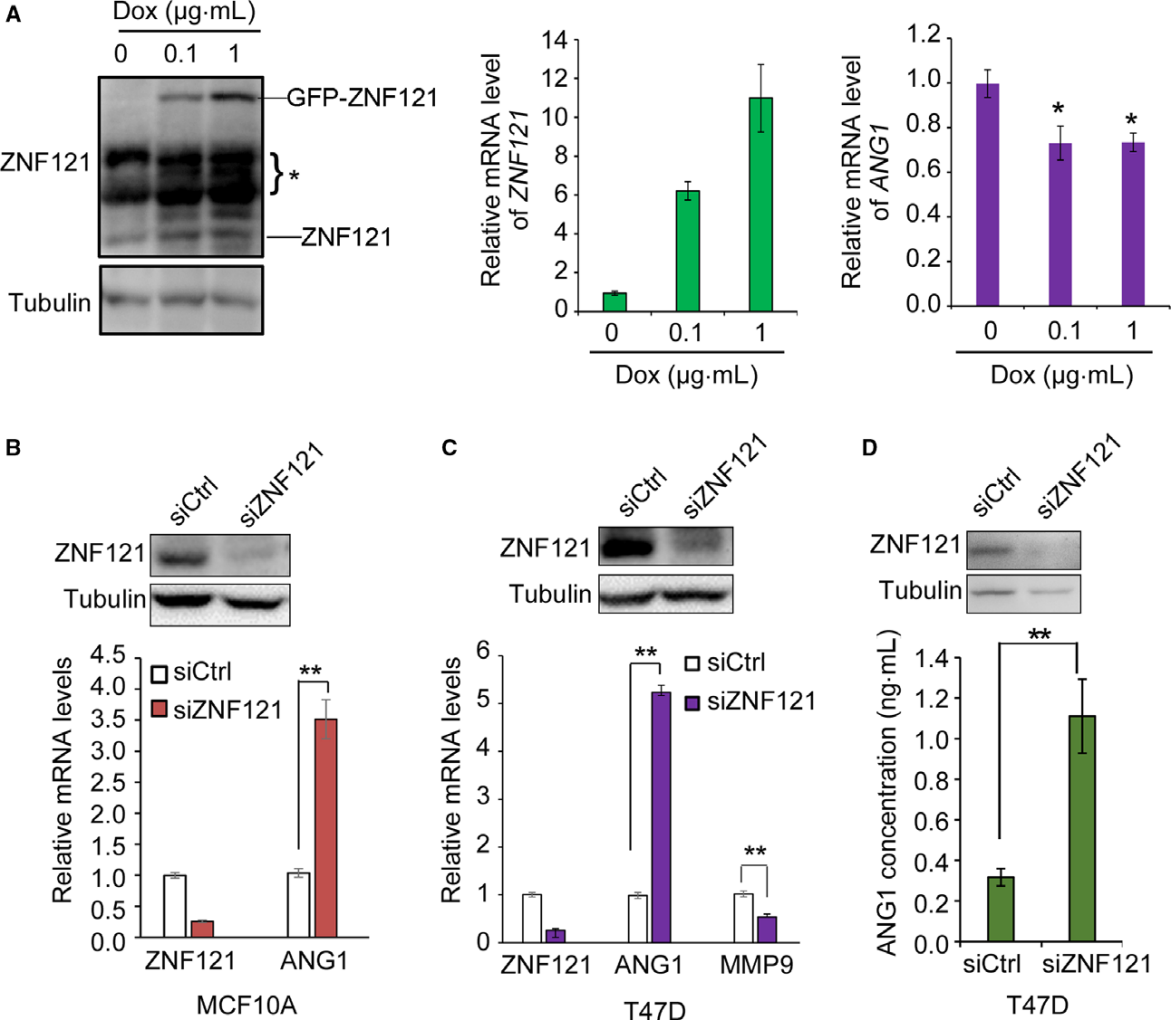
ZNF121 regulates the expression of ANG1 in MCF10A and T‐47D cells. (A) Overexpression of *ZNF121* reduces the mRNA level of *ANG1*. Conditional MCF10A cells were treated with different concentrations of Dox for 36 h. The cells were harvested both for WB with ZNF121 antibody or RNA isolation and qRT‐PCR with primers for *ZNF121*,* ANG1*. The protein level of tubulin served as the loading control. *, nonspecific bands. *ZNF121* knockdown increases the mRNA level of *ANG1* in MCF10A (B) and T‐47D (C) cells. Cells were transfected with control siRNA (siCtrl) or siRNAs targeting ZNF121 (siZNF121) for 48 h. Then, the cells were harvested for WB with ZNF121 antibody, or for RNA isolation and qRT‐PCR with primers for *ZNF121*,* ANG1*. (D) *ZNF121* knockdown in T‐47D cells increased the level of ANG1 protein in the medium. The efficiency of *ZNF121* siRNAs was confirmed by WB. For A‐D, data represent Mean ± SD (*n* = 3). Two‐tailed Student's *t*‐test, *P < 0.05; **P < 0.01.

Zinc finger and BRCA1‐interacting protein with a KRAB domain 1 and BRCA1 regulate the expression of a group of genes involved in human cancer by forming a complex with CtIP in MCF10A cells 11, 12, 13. After confirming the interactions of ZNF121 with ZBRK1 and BRCA1, we tested whether ZNF121 also regulates the expression of the common target genes of ZBRK1 and BRCA1, such as *ANG1*. We used a Tet‐On inducible system to express *ZNF121* in MCF10A breast epithelial cells. In this system, the ZNF121 coding region was fused with GFP, and the expression was successfully induced by Dox at both the RNA and protein levels (Fig. 3A). The mRNA level of *ANG1* was significantly reduced by *ZNF121* overexpression (Fig. 3A). Consistently, knockdown of *ZNF121* by RNAi significantly increased *ANG1* mRNA level in MCF10A cells (Fig. 3B). Similarly, *ZNF121* knockdown also increased the expression of *ANG1* in T‐47D breast cancer cells while the expression of matrix metallopeptidase 9 (*MMP9)*, another ZBRK1 target gene whose expression is independent of BRCA1, was decreased 16 (Fig. 3C). Because the ANG1 protein is a secreted factor, we examined the level of ANG1 protein in the cell culture medium using an ELISA. *ZNF121* knockdown in T‐47D cells also significantly increased ANG1 protein level in the medium (Fig. 3D). These results suggest that ZNF121 plays a role in the regulation of ANG1 expression in breast epithelial cells including breast cancer cells.

### ZNF121, ZBRK1, and BRCA1 coordinate to regulate the expression of ANG1

**Figure 4:**
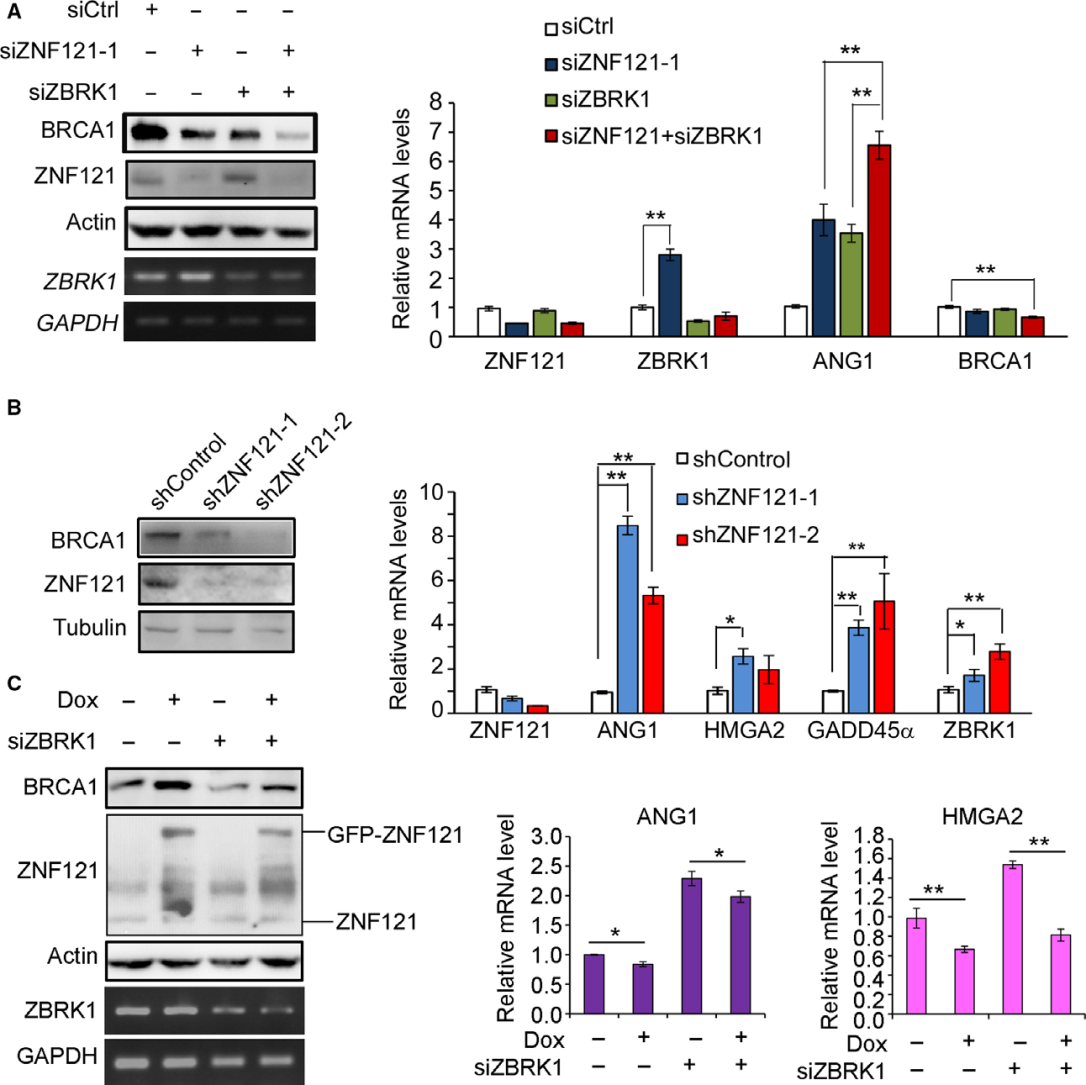
Cooperative regulation of BRCA1 and *ANG1* by ZNF121 and ZBRK1. (A) T‐47D cells were transfected with the indicated siRNAs, and 48 h later, the cells were harvested both for WB with antibodies for ZNF121 and BRCA1 and for semiquantitative PCR and qRT‐PCR with the indicated primers. The level of actin protein or *GAPDH* mRNA served as the internal control. (B) Stable knockdown of ZNF121 increases the common target genes of ZBRK1 and BRCA1 in T‐47D breast cancer cells. T‐47D cells expressing control shRNA (shControl) or ZNF121 shRNAs (shZNF121‐1, ‐2) were prepared for RNA extraction and then qRT‐PCR with the indicated primers (right). The efficiency of *ZNF121* shRNAs was confirmed by WB with ZNF121 antibody, and the protein level of BRCA1 was also examined (left). (C) Conditional MCF10A cells were respectively transfected with siCtrl or siRNA for ZBRK1 (siZBRK1) either in the presence or absence of Dox. Forty‐eight hours after transfection, the cells were harvested both for WB and semiquantitative PCR and qRT‐PCR with the indicated antibodies or primers. For A‐C, data represent Mean ± SD (*n* = 3). Two‐tailed Student's *t*‐test, *P < 0.05; **P < 0.01.

Zinc finger and BRCA1‐interacting protein with a KRAB domain 1 has been previously shown to regulate its downstream genes by directly binding to gene promoters. Taken together with the findings of ZNF121‐ZBRK1 interaction and ZNF121 regulation of *ANG1* expression, we tested whether the regulation of *ANG1* by ZNF121 depends on ZBRK1. We found that in T47‐D cells, knockdown of either *ZNF121* or *ZBRK1* increased *ANG1* mRNA expression, and an additive effect was observed when both *ZNF121* and *ZBRK1* were knocked down simultaneously (Fig. 4A), suggesting that ZNF121 and ZBRK1 regulate the expression of *ANG1* cooperatively. Unexpectedly, knockdown of *ZNF121* significantly increased the mRNA level of *ZBRK1* (Fig. 4A). Surprisingly, knockdown of either *ZNF121* or *ZBRK1* decreased the expression level of BRCA1, and their dual knockdown showed an additive effect, even though the decrease in mRNA level of *BRCA1* was not as significant as in the protein level (Fig. 4A). Similar changes were detected in MCF10A cells (data not shown). We also used another siRNA to knock down *ZNF121*, and similar effects were detected on the expression of BRCA1 protein and *ZBRK1* mRNA in both T‐47D cells and MCF10A cells (data not shown). The effects of ZNF121 knockdown on the expression of ANG1, ZBRK1, and BRCA1 were also confirmed in T‐47D cells expressing different ZNF121 shRNAs (Fig. 4B). In addition, we also found ZNF121 stable knockdown increased the expression of two other BRCA1 and ZBRK1 targets, HMGA2 and GADD45α (Fig. 4B).

We studied the cooperation between ZNF121 and ZBRK1 in the regulation of *ANG1* expression in MCF10A cells with Tet‐On inducible expression of ZNF121. As expected, induction of ZNF121 expression indeed increased the protein level of BRCA1 and decreased the mRNA level of *ANG1* in these cells (Fig. 4C). The inhibitory effect of ZNF121 overexpression on the mRNA expression of both *ANG1* was not affected by the knockdown of *ZBRK1* (Fig. 4C). In addition, we also found ZNF121 overexpression inhibited the mRNA level of *HMGA2*, an observation which was not affected by ZBRK1 knockdown (Fig. 4C).

## Discussion

Regulatory interactions between tumor suppressors and oncogenes create and maintain different cellular activities. For example, as one of the most potent oncogenes, MYC was found to bind to and regulate BRCA1 to control gene expression 17, 18, 19.

In this study, we performed a yeast two‐hybrid screen to identify potential ZNF121‐interacting proteins and focused on one such protein, ZBRK1, given its established interaction with the BRCA1 breast cancer gene and its role in human cancer development 16, 20. In addition, our previous study suggests that *ZNF121* is an oncogene 6. We confirmed the interaction between ZNF121 and ZBRK1 through the yeast two‐hybrid assay, combination of co‐IP and western blotting, and mapping of the ZBRK1 protein region that mediates its interaction with ZNF121 (Fig. 1).

We demonstrated that ZNF121 interacts with BRCA1 and regulates the expression of BRCA1 and two common target genes of BRCA1 and ZBRK1 (Figs. 2, 3, 4). Taken together with previous studies that have demonstrated the interaction between ZBRK1 and BRCA1 in gene regulation, these results suggest that ZNF121 also cooperates with ZBRK1 and BRCA1 in the regulation of their common target genes. Indeed, transient knockdown of *ZNF121* significantly promoted the expression of *ANG* in both the T‐47D breast cancer cell line and the MCF10A non‐neoplastic human breast epithelial cell line (Fig. 3). In MCF10A cells, stable overexpression of *ZNF121* inhibited the expression of *ANG1* and *HMGA2* (Figs 3 and 4). In addition, in T‐47D cells when ZNF121 was stably knocked down using shRNA, not only mRNA levels of both *ANG1* and *HMGA2* but also the expression of *GADD45α* were increased in comparison to the control cells (Fig. 4B). ZNF121 is thus likely another member of the protein complex composed of ZBRK1/BRCA1/CtIP.

Although it remains to be tested whether ZNF121 interacts with CtIP and whether ZNF121 is indeed a member of the ZBRK1/BRCA1/CtIP complex, the relationships among these proteins appear to be more complicated than expected. For example, knockdown of either ZNF121 or ZBRK1 led to reduced BRCA1 protein expression, and knockdown of both ZNF121 and ZBRK1 demonstrated an additive effect. However, knockdown of ZBRK1 appeared to erase the effect of ZNF121 on the expression of ANG1 and HMGA2, two common target genes of ZBRK1 and BRCA1 (Fig. 4C). It is possible that there are different complexes involving these proteins.

Interestingly, we found that *ZNF121* knockdown increased the level of *ZBRK1* mRNA, but we were unable to detect a change at the protein level due to the lack of a suitable antibody. It is thus unknown whether a direct regulatory effect or a feedback effect is responsible for the potential effect of ZNF121 on ZBRK1 expression. As for the regulation of BRCA1 by ZNF121, we propose a mechanism that is dependent on protein interaction, because the change in *BRCA1* mRNA caused by ZNF121 alteration was not always consistent with the change in BRCA1 protein expression. This possibility is in agreement with our unpublished data in which the reduction in BRCA1 protein by the knockdown of ZNF121 in T‐47D cells could be partly blocked by proteasome inhibitor treatment.

From the perspective of function, we propose that ZNF121 likely plays a role in tumor angiogenesis because of the increase in ANG1 protein in the medium of T‐47D cells with the knockdown of *ZNF121*. Considering that ZNF121 is overexpressed in breast cancer and knockdown of ZNF121 inhibits cell proliferation 6, it seems somewhat surprising that knockdown of ZNF121 also increased the expression of ANG1, an angiogenesis modulator. Nevertheless, enhanced expression of ANG1 has been shown to inhibit tumorigenesis 21, 22, which supports the hypothesis that ZNF121 affects tumorigenesis by regulating both MYC and ANG1.

## Author contributions

AL and JD conceived and designed the study. AL performed most of the experiments, analyzed the data, and drafted the manuscript. KZ and YZ contributed to construction and purification of plasmids. ZZ contributed to yeast two‐hybrid assay. LF contributed to the preparation of most reagents. JD supervised the project and finalized the manuscript.

## Conflict of interest

The authors declare no conflict of interest.
